# Two new spider species of the genus *Chrysso* O. P.-Cambridge, 1882 (Araneae, Theridiidae) in Hainan Island, China

**DOI:** 10.3897/zookeys.190.2924

**Published:** 2012-05-04

**Authors:** Bao-Shi Zhang, Feng Zhang

**Affiliations:** 1 Baoding University, Baoding, 071051, P. R. China; 2 College of Life Sciences, Hebei University, Baoding, Hebei 071002, P. R. China

**Keywords:** *Chrysso*, taxonomy, new species, newly recorded, China

## Abstract

Two new spider species of the genus *Chrysso* O. P.-Cambridge, 1882 are reported from Hainan Island, China, *Chrysso bifurca***sp. n.** (male, female) and *Chrysso bicuspidata***sp.** **n.** (male, female). *Chrysso bimaculata* Yoshida, 1998is recorded from China for the first time.

## Introduction

The genus *Chrysso* was erected by O. P.-Cambridge (1882). It was regarded as a junior synonymy of *Theridion* Walckenaer, 1805, and removed by Bryant (1940). Levi and Levi (1962) considered this genus as a senior synonym of *Arctachaea* Levi, 1957, *Argyroaster* Yaginuma, 1958 and *Meotipa* Simon, 1894, but Deeleman-Reinhold (2009) removed the genus *Meotipa* from the synonymy of *Chrysso*. Until now no large revision of this genus has been done. However, Agnarsson (2004) listed 10 autapomorphies of the *Chrysso* in his phylogeny, these characteristics are putative *Chrysso* synapomorphies.

Currently 62 *Chrysso* species are reported, mostly from America and Asia (Levi 1957; Levi and Levi 1962; Barrion and Litsinger 1995; Miller and Agnarsson 2005; Gonzaga et al. 2006; Yoshida 2009; Siliwal 2009; Platnick 2012), among them, 22 species are known from China. 94 species out of 24 genera are known from Hainan Island, China (Zhu 1998; Song et al. 1999; Yoshida et al. 2000; Tang et al. 2003; Song et al. 2006), including six *Chrysso* species: *Chrysso cyclocera* Zhu, 1998, *Chrysso trispinula* Zhu, 1998, *Chrysso trimaculata* Zhu, Zhang & Xu, 1991, *Chrysso scintillans* (Thorell, 1895), *Chrysso pulcherrima* (Mello-Leitão, 1917) and *Chrysso nigra* (O. P.-Cambridge, 1880).

During the examination of spider specimens collected from 2007 to 2009 in Hainan Island, China, two new species, *Chrysso bifurca* sp. n. and *Chrysso bicuspidata* sp. n. were recognized and are described here. *Chrysso bimaculata* Yoshida, 1998, known from Japan previously, is newly recorded from Hainan, China.

## Material and methods

All specimens were kept in 75% ethanol and examined, drawn and measured under a Tech XTL-II stereomicroscope equipped with an Abbe drawing device. Carapace length was measured medially from the anterior margin to the rear margin of the carapace. Eye sizes were measured as the maximum diameter of the lens in dorsal or frontal view. MOA length was measured medially from the anterior margin to the rear margin of MOA. Leg measurements are given as: total length (femur, patella, tibia, metatarsus, tarsus). Epigynes were cleared in warm potassium hydroxide (KOH) and transferred to 75% ethanol for drawing. The labeling of the palpal sclerites is adopted following Agnarsson (2004). All measurements are in millimeters. All specimens studied are deposited in the Museum of Hebei University (MHBU), Baoding, China.

### Abbreviations

AER anterior eye row

ALE anterior lateral eyes

AME anterior median eyes

C conductor

CD copulatory ducts

E embolus

FD fertilization ducts

MA median apophysis

MOA median ocular area

PER posterior eye row

PLE posterior lateral eyes

PME posterior median eyes

S spermathecae

ST subtegulum

T tegulum

TTA theridiid tegular apophysis

## Taxonomy

### Chrysso bifurca sp. n.

urn:lsid:zoobank.org:act:5B93397C-1884-4E05-9738-5B5A003645A6

http://species-id.net/wiki/Chrysso_bifurca

Figs 1–6

#### Type material.

**Holotype ♂, CHINA, Hainan Island:** Jianfengling Mountain (19°07'N, 109°13'E), 29 May 2009, C. Zhang leg. **Paratypes:** 2 ♀, same data as holotype; 1 ♀, Limu Mountain (19°10'N, 109°39'E), 20 August 2007, C. Zhang leg.; 3 ♂, 12 ♀, Limu Mountain, 20 November 2008, G.X. Han leg.

**Figures 1–6.**
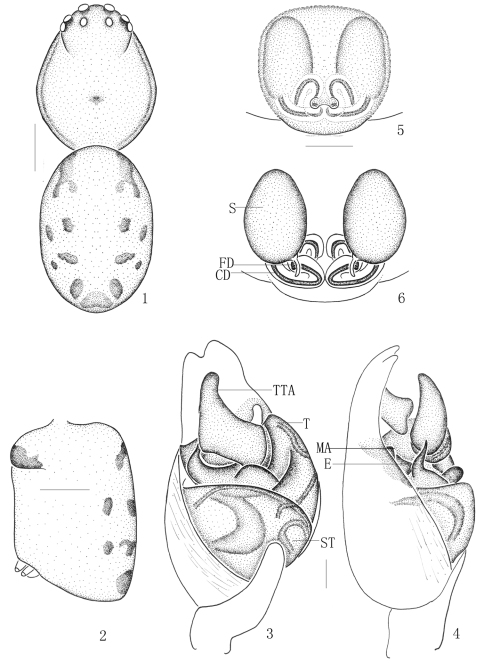
*Chrysso bifurca* sp. n., **1–4** male holotype **1** body, dorsal view **2** abdomen, lateral view **3** male left palp, ventral view **4** same, prolateral view **5–6** female paratype **5** epigynum, ventral view **6** vulva, dorsal view. Scale bars: 0.5 mm (**1–2**); 0.1 mm (**3–6**).

**Diagnosis.** Males can be distinguished from other *Chrysso* species by the following characters: apex of cymbium bifurcate; embolus short and thin; theridiid tegular apophysis erect, with obtuse apex (Figs 3–4). Females differ from other *Chrysso* species by the bigger and oval spermathecae, and the longer and winding copulatory ducts, differ from *Chrysso octomaculata* (Bösenberg & Strand, 1906) by the process of copulatory ducts (Fig. 6).

#### Etymology.

The species name refers to bifurcate apex of cymbium.

#### Description.

Male (holotype). Total length 2.70: cephalothorax 1.26 long, 1.08 wide; abdomen 1.44 long, 0.99 wide, 0.90 high. Carapace yellow, longer than wide, lateral margin with thin black striations. Only anterior part with several gray setae. Cervical groove distinct. Median furrow almost triangle, yellowish. Each eye with a black ring. Both the eye rows recurved from dorsal view (Fig. 1). Diameters of eyes: AME 0.12, ALE 0.10, PME 0.10, PLE 0.10. Interdistances of eyes: AME–AME 0.10, AME–ALE 0.08, ALE–ALE 0.64, PME–PME 0.10, PME–PLE 0.14, PLE–PLE 0.66, ALE and PLE closed to each other. MOA long 0.29, front width 0.34, back width 0.31. Clypeus 0.32 high, yellow and furnished with few short gray setae. Chelicerae armed with sparse gray setae, promargin with 2 teeth, fangs short and thin. Anterior margin of endites armed with gray scopula. Labium armed with sparse long black setae. Sternum furnished with sparse black setae, its anterior margin lightly procurved. Legs yellow, patella, metatarsus and the base of femur and tibia reddish-brown. Measurements of legs: leg I 9.44 (2.52, 0.59, 2.55, 2.88, 0.90), II 4.38 (1.49, 0.45, 1.13, 0.90, 0.41), III 3.16 (0.90, 0.36, 0.59, 0.90, 0.41), IV 5.09 (1.58, 0.36, 1.17, 1.44, 0.54). Leg formula: 1423.

Abdomen oval, longer than wide and armed with brown setae. Dorsum yellowish, armed with six pairs of irregular black patches, posterior with a median black patch (Figs 1–2). Venter yellowish, with a black median patch. Anal tubercle yellow. Spinnerets yellow.

Male palp with long cymbium, apical part of cymbium bifurcate; embolus short, base with a lunate process, distal part thin; theridiid tegular apophysis erect, with wide base, apex thin and obtuse; conductor lying behind theridiid tegular apophysis (Figs 3–4).

Female (one paratype from Jianfengling Mountain) total length 3.33: cephalothorax 0.95 long, 0.94 wide; abdomen 2.34 long, 1.62 wide, 1.58 high. Diameters of eyes: AME 0.12, ALE 0.10, PME 0.10, PLE 0.10. Interdistances of eyes: AME–AME 0.13, AME–ALE 0.09, ALE–ALE 0.59, PME–PME 0.14, PME–PLE 0.16, PLE–PLE 0.61, ALE and PLE closed to each other. MOA long 0.27, front width 0.30, back width 0.31. Clypeus 0.32 high. Measurements of legs: leg I10.81 (3.24, 0.63, 2.70, 3.29, 0.95), II 6.44 (2.03, 0.54, 1.44, 1.80, 0.63), III 3.52 (1.13, 0.41, 0.63, 0.90, 0.45), IV 7.08 (1.94, 0.54, 2.21, 1.80, 0.59). Leg formula: 1423. Other characters as in holotype.

Female genitalia lightly sclerotized, posterior part with a kidney-shaped atrium; spermathecae big, oval; copulatory ducts long, thick, winding and connected with spermathecae from posterior part (Figs 5–6).

The new species with putative *Chrysso* synapomorphies as follow:(1) carapace pars stridens irregular; (2) abdomen extending beyond spinnerets; (3) cymbial hood of male palp broad; (4) median apophysis of male palp with distinct apophysis; (5) palpal claw of female dentition sparse.

#### Variation.

The lateral part of dorsal abdomen of some females examined with eight to eleven pairs of irregular black patches. Males total body length from 2.64–2.80, female total length from 3.28–3.35.

#### Distribution.

China (Hainan).

### Chrysso bicuspidata sp. n.

urn:lsid:zoobank.org:act:660AA46B-0F72-4257-A1FC-8B734804876B

http://species-id.net/wiki/Chrysso_bicuspidata

Figs 7–12

#### Type material.

**Holotype ♂, CHINA, Hainan Island:** Jianfengling Mountain, 29 May 2009, C. Zhang leg. **Paratypes:** 2 ♀, same data as holotype; 3 ♀, Jianfengling Mountain, 12 November 2008, G.X. Han leg.; 3 ♂, 1 ♀, Bawangling Mountain (19°07'N, 109°04'E), 25 May 2009, C. Zhang leg.; 5 ♀, Bawangling Mountain, 7 November 2008, G.X. Han leg.; 2 ♂, 1 ♀, Diaoluo Mountain (18°45'N, 109°45'E), 6 June 2009, C. Zhang leg.; 1 ♂, 2 ♀, Limu Mountain, 19 August 2007, F. Zhang leg.

**Figures 7–12.**
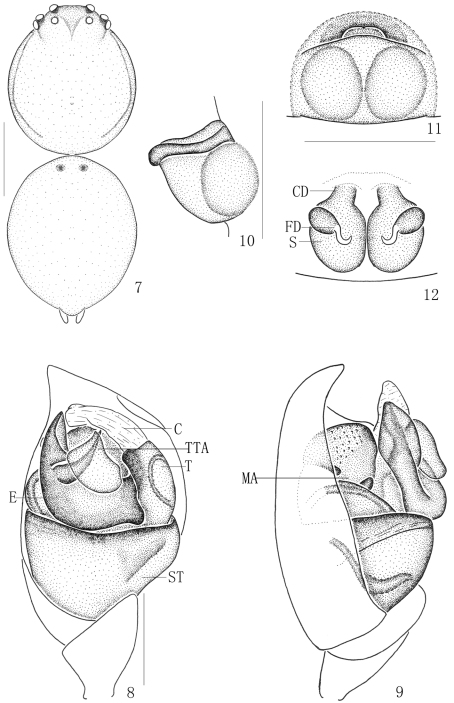
*Chrysso bicuspidata* sp. n., **7–9** female holotype **7** body, dorsal view **8** male left palp, ventral view **9** same, prolateral view **10–12** female paratype **10** epigynum, lateral view **11** same, ventral view **12** vulva, dorsal view. Scale bars: 0.5 mm (**7**); 0.1 mm (**8–12**).

#### Diagnosis.

Males can be distinguished from most *Chrysso* species by membranous conductor, wider embolus and acuate apex of cymbium. And it resembles *Chrysso cyclocera* Zhu, 1998 and *Chrysso oxycera* Zhu & Song, 1993 in the general shape of the palpal organ, but differs from them by the wider embolus (Figs 8–9). Females differ from all other *Chrysso* species except *Chrysso viridiventris* Yoshida, 1996 by the epigynum with a big atrium and a large posterior lobe. It differs from *Chrysso viridiventris* by the bigger spermathecae and shorter copulatory ducts (Figs 10–12).

#### Etymology.

The species name refers to the shape of embolic tip.

#### Description.

Male (holotype). Total length 1.84: cephalothorax 0.92 long, 0.71 wide; abdomen 0.92 long, 0.82 wide, 0.92 high. Carapace longer than wide, yellowish, lateral margin with thin black striations, anterior median with a triangular black patch. Only anterior part with several gray setae. Cervical groove yellowish. Median furrow yellowish, almost triangle. Each eye with a black ring. Both the eye rows recurved from dorsal view (Fig. 7). Diameters of eyes: AME 0.05, ALE 0.04, PME 0.04, PLE 0.04. Interdistances of eyes: AME–AME 0.10, AME–ALE 0.03, ALE–ALE 0.33, PME–PME 0.10, PME–PLE 0.08, PLE–PLE 0.38, ALE and PLE closed to each other. MOA long 0.13, front width 0.18, back width 0.18. Clypeus 0.20 high and furnished with few short gray setae. Chelicerae armed with sparse gray setae, promargin with 2 teeth, fangs short and thin. Endites, labium and sternum yellowish. Anterior margin of endites armed with gray scopula. Sternum furnished with sparse gray setae. Legs yellowish, the end of tibia with gray spots. Measurements of legs: leg I 7.14 (1.94, 0.41, 1.94, 1.63, 1.22), II 3.69 (1.33, 0.31, 0.82, 0.82, 0.41), III 2.14 (0.71, 0.20, 0.31, 0.61, 0.31), IV 3.86 (1.12, 0.31, 0.82, 1.20, 0.41). Leg formula: 1423.

Abdomen oval, longer than wide and armed with brown setae. Dorsum yellowish, anterior part with a pair of black patches (Fig. 7). Venter yellowish. Spinnerets yellowish.

Apical cymbium of male palp acuate; embolus big, thick, and end with a thin ramus; conductor membranous, falciform from ventral view; apex of median apophysis with some small tubers (Figs 8–9).

Female (one paratype from Limu Mountain) total length 2.32: cephalothorax 0.92 long, 0.71 wide; abdomen 1.43 long, 1.22 wide, 1.43 high. Diameters of eyes: AME 0.05, ALE 0.05, PME 0.03, PLE 0.05. Interdistances of eyes: AME–AME 0.05, AME–ALE 0.02, ALE–ALE 0.30, PME–PME 0.09, PME–PLE 0.08, PLE–PLE 0.33, ALE and PLE closed to each other. MOA long 0.13, front width 0.15, back width 0.15. Clypeus 0.30 high. Measurements of legs: leg I 7.75 (2.24, 0.51, 1.94, 2.35, 0.71), II 3.59 (1.24, 0.31, 0.71, 0.92, 0.41), III 2.04 (0.71, 0.20, 0.31, 0.51, 0.31), IV 3.79 (1.24, 0.31, 0.81, 1.02, 0.41). Leg formula: 1423. Dorsal abdomen yellowish. Other characters as in holotype.

Female genitalia lightly sclerotized, and with a circular atrium and a large posterior lobe; spermathecae big, oval; copulatory ducts short, thick and connected with spermathecae from anterior part; each fertilization duct with a global head (Figs 10–12).

The new species with putative *Chrysso* synapomorphies as follow:(1) carapace pars stridens smooth; (2) abdomen extending beyond spinnerets; (3) cymbial hood of male palp broad; (4) subconductor of male palp present; (5) median apophysis of male palp with apophysis; (6) palpal claw of female dentition sparse; (7) anterior margin of female genital atrium medially acute.

#### Variation.

The anterior part of dorsal abdomen of some species examined with a pair of black patches, some species without. Males total body length from 1.73–1.88, females total length from 2.22–2.35.

#### Distribution.

China (Hainan).

### Chrysso bimaculata

Yoshida, 1998

http://species-id.net/wiki/Chrysso_bimaculata

Figs 13–17

Chrysso bimaculata Yoshida, 1998: 105, f. 1–6; Yoshida 2003: 125, f. 330–335; Yoshida 2009: 378, f. 203–204.

#### Material examined.

**CHINA, Hainan Island:** 1 ♂, 1 ♀, Jianfengling Mountain, 31 May 2009, C. Zhang leg.; 1 ♀, Bawangling Mountain, 6 November 2008, G.X. Han leg.; 1 ♀, Bawangling Mountain, 25 May 2009, C. Zhang leg.; 5 ♀, Limu Mountain, 29 August 2007, G.X. Han leg.; 1 ♂, 3 ♀, Limu Mountain, 21 November 2008, G.X. Han leg.

**Figures 13–17.**
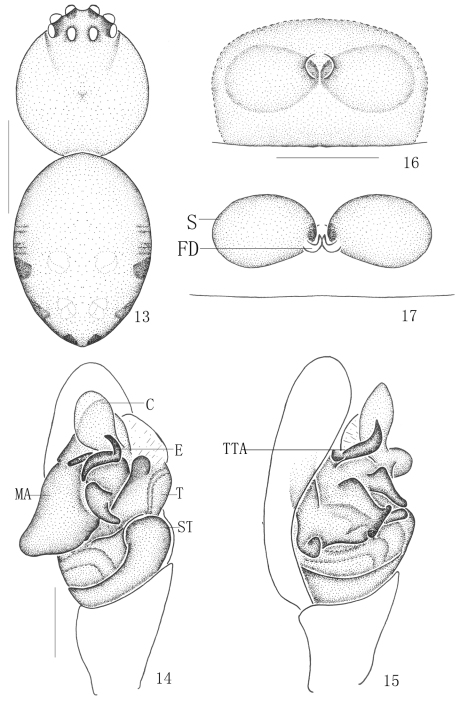
*Chrysso bimaculata* Yoshida, 1998 **13–15** male **13** body, dorsal view **14** male left palp, ventral view **15** same, prolateral view **16–17** female **16** epigynum, ventral view **17** vulva, dorsal view. Scale bars: 0.5 mm (**13**); 0.1 mm (**14–17**).

#### Description.

Male (one specimen from Jianfengling Mountain) total length 1.40: cephalothorax 0.59 long, 0.54 wide; abdomen 0.81 long, 0.59 wide, 0.54 high. Carapace longer than wide, yellowish. Cervical groove yellowish. Median furrow yellowish and almost triangle. Each eye with a red ring. AER recurved and PER procurved from dorsal view (Fig. 13). Diameters of eyes: AME 0.08, ALE 0.07, PME 0.07, PLE 0.07. Interdistances of eyes: AME–AME 0.08, AME–ALE 0.07, ALE–ALE 0.34, PME–PME 0.08, PME–PLE 0.06, PLE–PLE 0.36, ALE and PLE closed to each other. MOA long 0.18, front width 0.18, back width 0.17. Clypeus 0.16 high and furnished with few short gray setae. Chelicerae armed with sparse black setae, promargin with 2 teeth. Endites, labium and sternum yellowish. Anterior margin of endites armed with gray scopula. Sternum furnished with sparse black setae. Legs yellow, the end of tibia yellow brown. Measurements of legs: leg I 5.10 (1.44, 0.36, 1.22, 1.58, 0.50), II 3.07 (0.99, 0.32, 0.63, 0.77, 0.36), III 1.81 (0.50, 0.23, 0.45, 0.36, 0.27), IV 3.20 (1.17, 0.27, 0.63, 0.77, 0.36). Leg formula: 1423.

Abdomen oval, armed with gray setae. Dorsum yellowish, posterior part with two pairs of white patches, lateral part armed with three pairs of black patches and several black stripes (Fig. 13). Venter yellowish. Spinnerets yellowish.

Embolus of male palp small, thin and wind; conductor wind, membranous; median apophysis big, posterior part with a small tuber from prolateral view (Figs 14–15).

Female (one specimen from Jianfengling Mountain) total length 1.80: cephalothorax 0.63 long, 0.54 wide; abdomen 1.08 long, 0.95 wide, 1.04 high. Diameters of eyes: AME 0.08, ALE 0.07, PME 0.07, PLE 0.07. Interdistances of eyes: AME–AME 0.09, AME–ALE 0.03, ALE–ALE 0.35, PME–PME 0.10, PME–PLE 0.07, PLE–PLE 0.38, ALE and PLE closed to each other. MOA long 0.17, front width 0.17, back width 0.20. Clypeus 0.16 high. Measurements of legs: leg I 4.73 (1.35, 0.27, 1.17, 1.44, 0.50), II 2.81 (0.90, 0.23, 0.59, 0.77, 0.32), III 1.95 (0.63, 0.18, 0.32, 0.50, 0.32), IV 2.94 (0.95, 0.27, 0.59, 0.77, 0.36). Leg formula: 1423. Other characters as in holotype.

Female genitalia lightly sclerotized, median part with a circular atrium; spermathecae oval; the copulatory ducts very short (Figs 16–17).

#### Variation.

The amount of white patches and black patches of dorsal abdomen variated from two pairs to three pairs. Males total body length of some species examined from 1.40–1.55, females total length from 1.68–1.82.

#### Distribution.

China (Hainan), Japan.

## Supplementary Material

XML Treatment for Chrysso bifurca

XML Treatment for Chrysso bicuspidata

XML Treatment for Chrysso bimaculata
